# One-Step Hydrothermal Synthesis of Zeolite X Powder from Natural Low-Grade Diatomite

**DOI:** 10.3390/ma11060906

**Published:** 2018-05-28

**Authors:** Guangyuan Yao, Jingjing Lei, Xiaoyu Zhang, Zhiming Sun, Shuilin Zheng

**Affiliations:** School of Chemical and Environmental Engineering, China University of Mining and Technology (Beijing), Beijing 100083, China; slxw.yao1990@hotmail.com (G.Y.); 18811568019@163.com (J.L.); zhangxiaoyukdbj@163.com (X.Z.)

**Keywords:** diatomite, zeolite X, hydrothermal method, calcium ion exchange capacity

## Abstract

Zeolite X powder was synthesized using natural low-grade diatomite as the main source of Si but only as a partial source of Al via a simple and green hydrothermal method. The microstructure and surface properties of the obtained samples were characterized by powder X-ray diffraction (XRD), scanning electron microscopy (SEM), wavelength dispersive X-ray fluorescence (XRF), calcium ion exchange capacity (CEC), thermogravimetric-differential thermal (TG-DTA) analysis, and N_2_ adsorption-desorption technique. The influence of various synthesis factors, including aging time and temperature, crystallization time and temperature, Na_2_O/SiO_2_ and H_2_O/Na_2_O ratio on the CEC of zeolite, were systematically investigated. The as-synthesized zeolite X with binary meso-microporous structure possessed remarkable thermal stability, high calcium ion exchange capacity of 248 mg/g and large surface area of 453 m^2^/g. In addition, the calcium ion exchange capacity of zeolite X was found to be mainly determined by the crystallization degree. In conclusion, the synthesized zeolite X using diatomite as a cost-effective raw material in this study has great potential for industrial application such as catalyst support and adsorbent.

## 1. Introduction

Zeolites are crystalline aluminosilicates built from TO_4_ tetrahedra (T = Si and Al) with excellent properties of high surface area, uniform and precise microporosity, shape selectivity, high ion-exchange capacity, strong Brønsted acidity and high thermal and hydrothermal stability [1]. Therefore, zeolites have been widely used in many environmental and other industrial applications, such as ion exchange [2,3,4,5], catalysts [6,7,8,9], membrane separations [10,11,12] and adsorbents [13,14,15,16,17].

The principle raw materials used for the synthesis of the zeolites are different sources of silica and alumina, which are usually composed of sodium silicates, sodium aluminate, aluminum salts or colloidal silica. However, traditional methods for synthesizing zeolites typically involve chemical reagents as starting materials or crystallization from a gel or clear solution under hydrothermal conditions, which have the disadvantages of high cost, excessive waste, and unfriendly nature to the environment. Therefore, many attempts are underway for economical synthesis of zeolites. In general, natural aluminosilicate and silicate minerals and industrial solid wastes have been explored as silica and/or alumina source because they are cost-effective precursors and can lead to reduction of the synthesis costs. Until now, There have been many studies on synthesizing zeolites from natural minerals such as kaolinite [18,19,20], bentonite [19], feldspar [19,21] and other precursors [22,23,24,25,26].

Although zeolites have been synthesized from the solid wastes, such as fly ash [27,28,29], rice husk ash [30] and coal gangue [22], the uncertainty in their supplies and the impurity in their components may limit their practical application. Therefore, direct synthesis of zeolites from natural aluminosilicate and silicate minerals with abundant reserves in the earth has been pursued because of its great potential in reducing the generation of hazardous wastes, saving energy, and possibly altering the properties of the resulting zeolites [31]. However, most aluminosilicate minerals are inactive, which restricted their practical application in the zeolite synthesis. Additionally, even after the thermal activation, only part of the aluminum-oxygen bonds can be broken, which means that just part of the Al_2_O_3_ and a small amount of SiO_2_ only can participate in the zeolite synthesis.

Diatomite is an interesting silica material because of its relatively low cost, large reserves and its highly reactive amorphous state derived from silica skeletons of diatoms. Being silica rich, diatomite serves as the silica source in the synthesis of zeolites, which is cost-effective. In addition, diatomite is amorphous and highly reactive and therefore, unnecessary to transform it into a reactive state as done with many crystalline minerals [18,19,20,21]. Furthermore, parts of secondary building units of minerals could be preserved in the synthesis process. Therefore, it is necessary to explore detailed research on the preparation and formation mechanisms of zeolites from diatomite.

In the present work, we report on the synthesis of zeolite X from natural low-grade diatomite with high crystallization degree under hydrothermal conditions. Additionally, the effect of factors such as aging time and temperature, crystallization time and temperature, Na_2_O/SiO_2_ and H_2_O/Na_2_O ratio on the synthesis of zeolite in the SiO_2_-Al_2_O_3_-Na_2_O-H_2_O system were systematically investigated. The crystallization degree of the products was evaluated by XRD, SEM and CEC analysis. Meanwhile, the formation mechanism was also explored and discussed. 

## 2. Experimental

### 2.1. Materials

Diatomite (Dt) is obtained from Linjiang City, Jilin Province, China. Its main chemical composition by wt % is: SiO_2_: 63.77%; Al_2_O_3_: 18.97%; Fe_2_O_3_: 1.48%; CaO: 0.48%; K_2_O: 0.16%; Na_2_O: 0.04%. It was grounded to a size smaller than 30 mesh and dried at 105 °C. Commercial zeolites X were purchased from Tianjin yuanli Reagent Co. (Tianjin yuanli, China). Sodium hydroxide, aluminum hydroxide (nordstrandite) and the other chemicals used in the experiments were purchased from Xilong Reagent Co. (Xilong, China). All chemicals were of analytical reagent grade and used without any further purification. Deionized water was used throughout this study.

### 2.2. Preparation of Zeolite X

The synthesis of zeolites from diatomite includes three processes as follows: gel formation, aging and crystallization. Initially, diatomite and Al(OH)_3_ were dispersed in NaOH solution under vigorous magnetic stirring to form a homogeneous dispersion. The amount of diatomite and Al(OH)_3_ were according to the 1.13 (molar ratio) of [Si/Al], and the amount of NaOH solution was according to the molar ratios of [Na_2_O/SiO_2_] and [H_2_O/Na_2_O]. Subsequently, the slurry was subjected to aging for 0–120 min at 30–60 °C. Then, the mixed solution was put into a Teflon-lined stainless steel autoclave. Finally, the container was closed and crystallized at 90–120 °C for 3–9 h. After that, the autoclave was cooled to room temperature naturally, and the samples were removed from the reactor, filtered, and washed with deionized water until the pH of the filtrate reached 6–7. Finally, the wet washed solids were dried overnight at 105 °C before further measurement and characterization.

### 2.3. Characterization

X-ray diffraction (XRD) analysis were performed on a D8 advance X-ray diffractometer (Bruker, Germany) equipped with Cu-Kα radiation (λ = 0.154056 nm) to identify the crystalline phase of the obtained X zeolite products. The samples were scanned in the 2θ range of 5° to 50° with a 0.02° step at a scanning speed of 4°/min. The surface morphology of the samples was observed by S-4800 scanning electron microscope (Hitachi, Japan). Nitrogen adsorption-desorption isotherms were measured at 77 K using an ASAP 2020 instrument (Micromeritics, Norcross, GA, USA), after evacuation of the samples at 350 °C for 4 h. The specific surface area (SBET) and microporous volume (Vmicro) were calculated using the BET and t-plot methods, respectively. Pore size distribution curves were calculated by Barrett-Joyner-Halenda (BJH) method. The crystallization behavior of zeolites as well as the thermal properties of the composites was monitored and evaluated using a Mettler TGA/DSC 1 SF/1382 equipment (NETZSCH, Germany). The TGA/DSC measurements were carried out in air flow with a heating rate of 5 °C/min from 25–900 °C. Chemical composition of the sample was determined using wavelength dispersive X-ray fluorescence spectrometry (XRF, Shimadzu, Japan) on a Shimadzu XRF-1800 apparatus. Calcium ion exchange capacity (CEC) was determined as follows [22]: Typically, 0.5 g of zeolite sample was poured into 500 mL of 0.005 M CaCl_2_ solution and the mixture was shaken for 20 min at 35 °C. Then the filtrate was analyzed by the addition of calconcarboxylic acid and EDTA to determine the CEC of the samples.

## 3. Results and Discussion

### 3.1. Starting Materials

**Figure 1:**
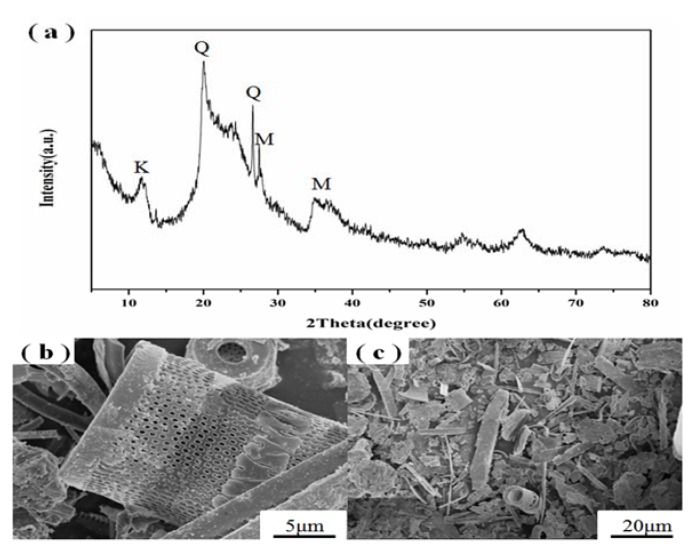
XRD pattern (**a**) and SEM images (**b**,**c**) of raw diatomite. Q = quartz; M = montmorillonite; K = kaolinite.

The XRD pattern (a) and SEM images (b,c) of raw diatomite are shown in Figure 1. According to the XRD pattern of diatomite, the broad reflection centered at 2θ = 15–30° was attributed to the amorphous silica, and the peaks at 2θ = 20.08° and 26.65° were ascribed to quartz. In addition, the peaks at 2θ = 11.88°, 27.35° and 35°–40° were characteristic to kaolinite-montmorillonite [32], which were the main Al source. As shown in Figure 1b,c, the diatomite exhibits highly porous cylinder-like or boat-like shape.

### 3.2. Effect of Crystallization

**Figure 2:**
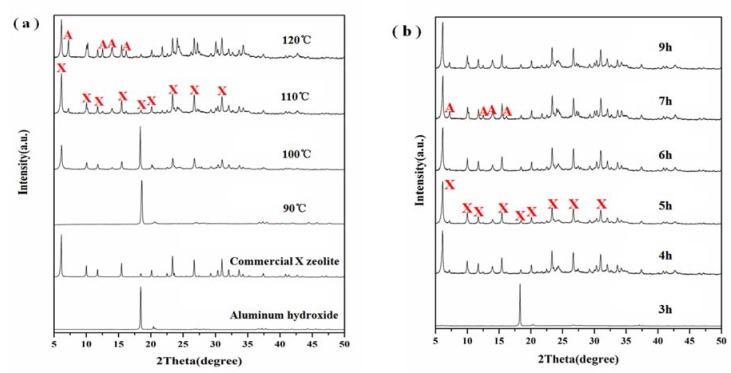
XRD patterns showing the effect of (**a**) crystallization temperature at crystallization time of 5 h and (**b**) crystallization time at crystallization temperature of 110 °C on zeolite formation.

Crystallization conditions are important parameters that control the crystallization of zeolites. The crystallization conditions could also change the autogenous pressure in the autoclave and may alter the structure of the resulting zeolites. Then, a batch of experiments were carried out under different crystallization temperatures and times, while the other preparation conditions including aging temperature and time, Na_2_O/SiO_2_ and H_2_O/Na_2_O ratio were kept constant. Namely: 30 °C of aging temperature, 60 min of aging time, 40 of H_2_O/Na_2_O ratio and 3.0 of Na_2_O/SiO_2_ ratio. The XRD patterns of samples with different crystallization temperatures and times are shown in Figure 2a,b, respectively. It can be seen from Figure 2a that the sample at a crystallization temperature of 90 °C showed only one strong diffraction peak at 2θ = 18.35°, which can be attributed to aluminum hydroxide indicating that diatomite was dissolved in the alkaline solution, but the silica did not react with aluminum hydroxide. When the temperature was raised to 100 °C, typical diffraction peaks of X zeolite (JCPDS 38-0237) can be seen at 2θ = 6.10°, 9.97°, 11.76°, 15.39°, 18.46°, 20.12°, 23.24°, 26.58° and 30.86° suggesting that zeolite X started to crystallize (Figure 2a). However, the diffraction peak of aluminum hydroxide can also be seen at 2θ = 18.35°, which suggested an incomplete reaction of aluminum hydroxide and silica from diatomite. Highly crystalline single phase zeolite X was formed as the crystallization temperature was raised to 110 °C. When the reaction temperature was further raised to 120 °C, almost pure phase of zeolite X was observed along with some zeolite A (JCPDS 43-0142). It indicated that the higher crystallization temperature of 120 °C caused the transformation of some zeolite X into zeolite A. As shown in Figure 2b, no zeolite X was obtained at crystallization time of 3 h at 110 °C. With the increase of crystallization time, the crystallization degree of zeolite X was enhanced. However, longer reaction time caused the transformation of zeolite X into zeolite A at 110 °C. Therefore, suitable crystallization temperature and time are essential for the formation of high purity zeolite X.

**Figure 3:**
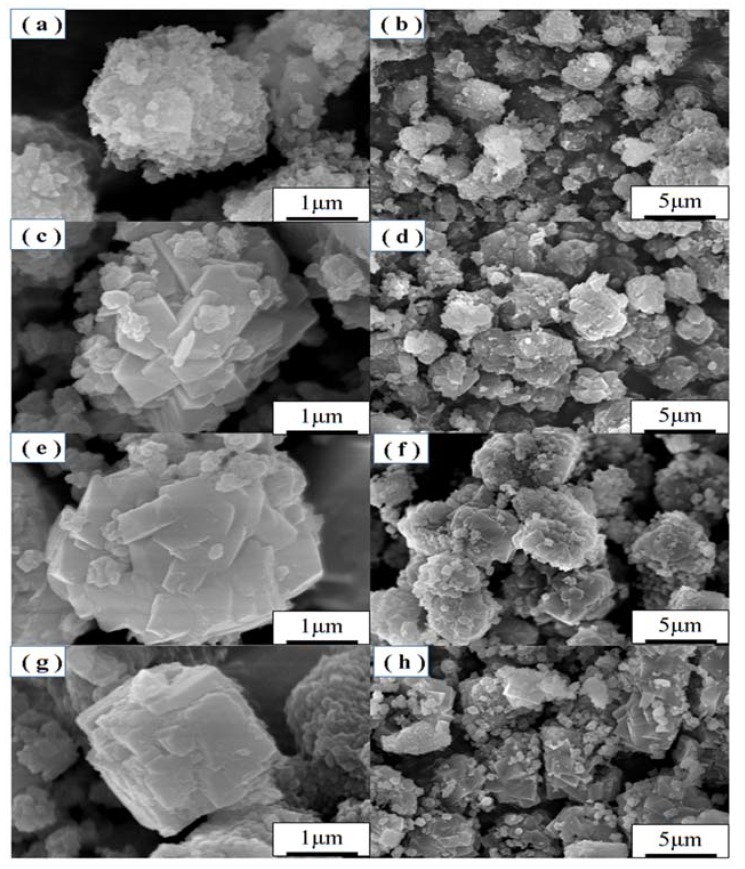
SEM images of zeolites obtained with crystallization temperature at (**a**,**b**) 90 °C, (**c**,**d**) 100 °C, (**e**,**f**) 110 °C and (**g**,**h**) 120 °C.

SEM images of samples obtained at different crystallization temperatures are shown in Figure 3. When the crystallization temperature was 90 °C, the reactants formed spherical aggregates of ill-defined particles. With the increase of crystallization temperature, the products gradually formed regular crystals with smooth faces. However, the crystallization temperature at 110 °C formed better octahedral crystal morphology along with uniform distribution of crystals (Figure 3e,f). In addition, amorphous material was hardly noticeable in the SEM images of as-synthesized zeolite X suggesting the formation of highly crystalline zeolite X. When the crystallization temperature was increased up to 120 °C, some cubic crystals appeared (Figure 3g,h), which could be attributed to the formation of zeolite A.

**Figure 4:**
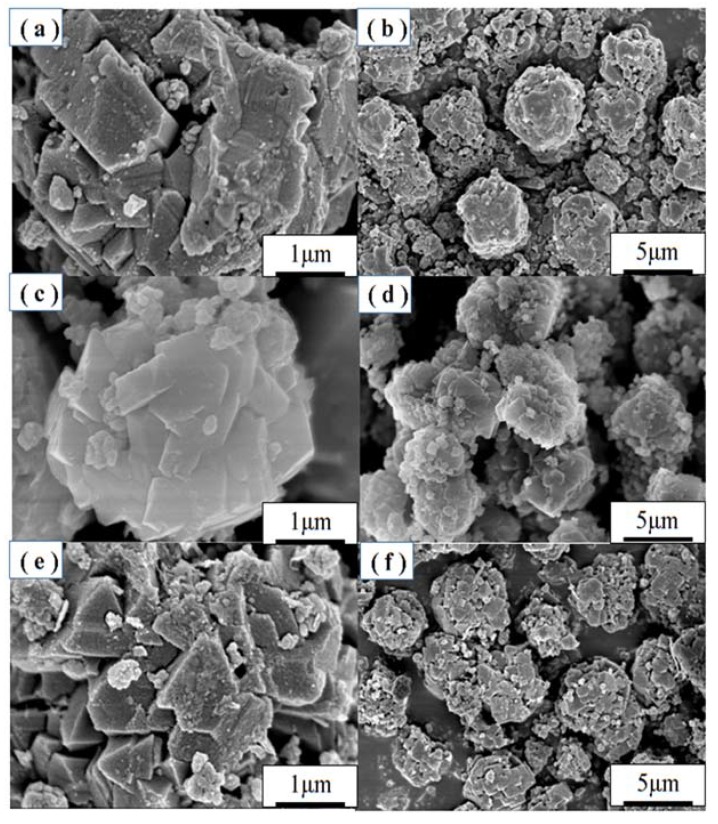
SEM images of zeolites obtained with crystallization time at (**a**,**b**) 4 h, (**c**,**d**) 5 h and (**e**,**f**) 6 h at 110 °C.

The morphologies of the samples prepared at different crystallization times at 110 °C are shown in Figure 4. When the crystallization time was 4 h, the synthesized products formed crystals with no distinct faces, and the particles were not uniform. When the crystallization time reached 5 h, the synthesized products formed regular crystals with smooth faces. When the crystallization time was up to 6 h, the synthesized products do not show smooth faces. The morphologies as determined by SEM with different crystallization temperatures and times are in accordance with the XRD results. When the crystallization temperature and time were 110 °C and 5 h, the prepared samples showed higher crystallization degree.

**Figure 5:**
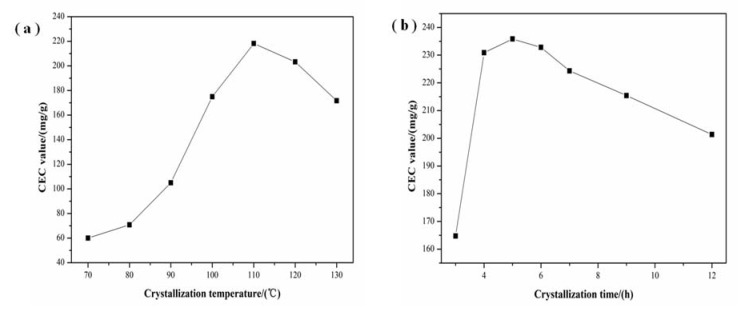
Effect of (**a**) crystallization temperature and (**b**) crystallization time on CEC.

The influence of crystallization temperature and time on the CEC is presented in Figure 5a,b. Increasing temperature of crystallization up to 110 °C increased the CEC but by further increasing the temperature up to 130 °C, the CEC of prepared samples gradually decreased (Figure 5a). Furthermore, the CEC of samples firstly increased with the increase of crystallization time and then gradually decreased when the time was over 5 h (Figure 5b). Combined with the analysis of SEM and XRD, it is concluded that the CEC increased because of the enhancement of crystallization degree of zeolite X.

### 3.3. Effect of Aging

**Figure 6:**
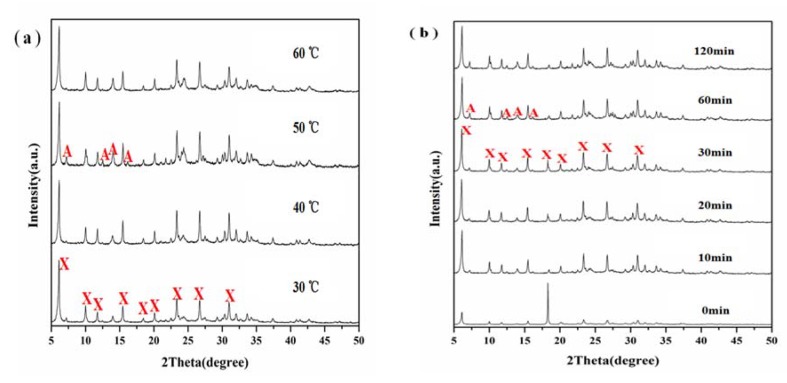
XRD patterns showing the effect of (**a**) aging temperature at aging time of 60 min and (**b**) aging time at aging temperature of 30 °C on zeolite formation after treatment at 110 °C and 5 h.

Aging also played an important role in the nucleation of amorphous gel. During this stage, the structure and composition of the silica-alumina gel changed along with the aging conditions. Meanwhile, the aluminosilicate species included in the gel phase were also transformed [33]. To investigate the effect of aging conditions on the structure of the products, a batch of experiments under different aging temperatures and times were carried out by keeping the crystallization temperature and time constant at 110 °C and 5 h, respectively, and the alkalinity of the base solutions at the initial values. The XRD patterns of samples prepared at different aging temperatures and times are shown in Figure 6a,b, respectively. The XRD peaks of the prepared products at different aging temperatures exhibited similar crystallinity of zeolite X (Figure 6a) indicating that aging temperature within this range played only a minor role, if any in the formation of zeolite X. However, the synthetic product without aging exhibited extremely weak diffraction peaks of zeolite X (Figure 6b). Also, there was a sharp diffraction peak at 2θ = 18.35°, which could be attributed to the unreacted aluminum hydroxide. The diffraction peaks of the prepared products at different aging times exhibited the same typical features of zeolite X with high crystallization degree. However, when the aging time was up to 60 and 120 min, some obvious diffraction peaks of zeolite A appeared indicating that the aging time plays a significant role in the formation of zeolite prepared from diatomite.

**Figure 7:**
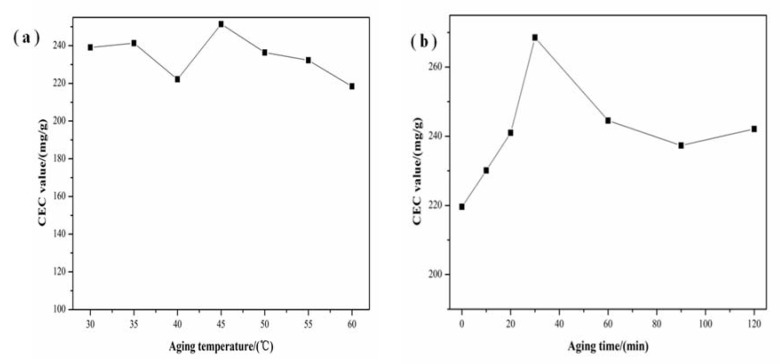
Effect of (**a**) aging temperature and (**b**) aging time on CEC of zeolites prepared upon hydrothermal treatment at 110 °C and 5 h.

The CEC values of samples with different aging temperatures and times are displayed in Figure 7a,b, respectively. As shown in Figure 7a, the CEC values of samples at different aging temperatures fluctuated little. The CEC values firstly increased with the increase of aging time and then gradually decreased when the time is over 30 min (Figure 7b). Hence, on the basis of XRD and CEC analysis, samples with high crystallization degree possessed high CEC value.

### 3.4. Effect of Alkalinity

**Figure 8:**
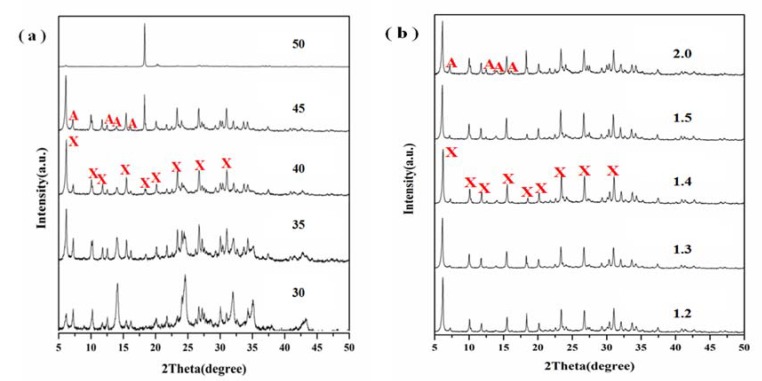
XRD patterns showing the effect of (**a**) H_2_O/Na_2_O ratio and (**b**) Na_2_O/SiO_2_ ratio on zeolite formation upon hydrothermal treatment at 110 °C and 5 h.

Another important parameter that controls the nucleation and crystal growth of zeolite is the alkalinity of the base solution, which includes H_2_O/Na_2_O and Na_2_O/SiO_2_ ratios [34]. Generally high alkaline concentration of the system could accelerate the dissolution of silicon and aluminum components in the precursor materials, which will shorten the induction period and nucleation time, and then speed up the crystallization rate. To investigate the effect of alkalinity on the structure of the products, a batch of experiments under different H_2_O/Na_2_O and Na_2_O/SiO_2_ ratios were carried out with the crystallization temperature and time of 110 °C and 5 h and the aging temperature and time of 30 °C and 30 min. The XRD patterns of samples with different H_2_O/Na_2_O and Na_2_O/SiO_2_ ratios are shown in Figure 8a,b, respectively. As the H_2_O/Na_2_O ratio increased, its crystallization degree was enhanced (Figure 8a). When the H_2_O/Na_2_O ratios were 40 and 45, the crystallization degree reached the maximum and there were also some zeolite A impurities. However, when the H_2_O/Na_2_O ratios were 45, a relatively sharp diffraction peak of aluminum hydroxide appeared (Figure 8a), which indicated that diatomite and aluminum hydroxide did not react completely. When the H_2_O/Na_2_O ratio was up to 50, there was only one sharp diffraction peak attributable to aluminum hydroxide. This reveals that higher H_2_O/Na_2_O ratios may inhibit the formation of zeolite X from diatomite, probably because low alkalinity reduces the dissolution of diatomite and results in a low conversion to zeolites. Figure 8b shows that the crystallization degree at different Na_2_O/SiO_2_ ratio was relatively high, which indicated that Na_2_O/SiO_2_ ratio within this range played minor effects in the formation of zeolite X.

**Figure 9:**
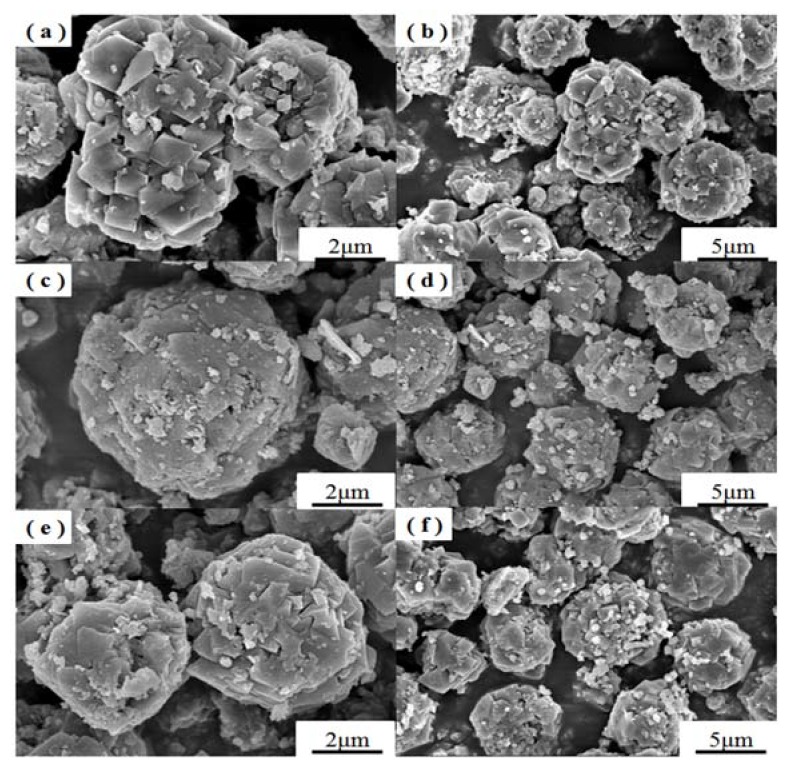
SEM images of zeolites obtained at Na_2_O/SiO_2_ ratio of (**a**,**b**) 1.3, (**c**,**d**) 1.4 and (**e**,**f**) 1.5 upon hydrothermal treatment at 110 °C and 5 h.

SEM images of samples with different Na_2_O/SiO_2_ ratios are presented in Figure 9. Figure 9 shows that all samples with Na_2_O/SiO_2_ ratios of 1.3, 1.4 and 1.5 exhibited high crystallinity with smooth faces. On the other hand, the sample with Na_2_O/SiO_2_ ratio of 1.4 exhibited better octahedral structure and uniform distribution.

**Figure 10:**
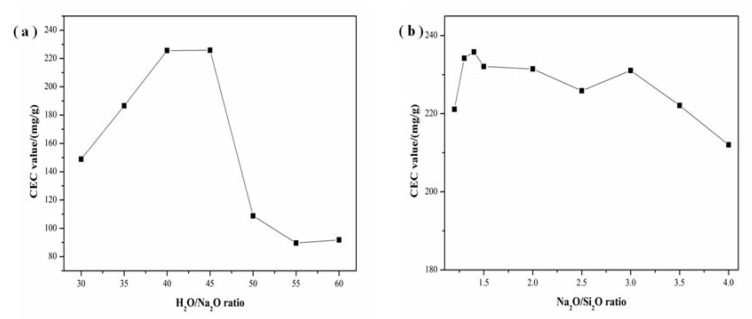
Effect of (**a**) H_2_O/Na_2_O ratio and (**b**) Na_2_O/SiO_2_ ratio on CEC upon hydrothermal treatment at 110 °C and 5 h.

The influence of H_2_O/Na_2_O and Na_2_O/SiO_2_ ratios on the CEC is shown in Figure 10a,b. As shown in Figure 10a, the increase of H_2_O/Na_2_O ratio resulted in the increase of the CEC. However, when the H_2_O/Na_2_O ratio was higher than 40, its CEC gradually decreased. It can be attributed to the low alkalinity, which reduces the dissolution of diatomite and results in a low conversion to zeolites. As displayed in Figure 10b, the CEC at different Na_2_O/SiO_2_ ratios maintained a high value and fluctuate only a little. It is obvious that higher crystallization degree led to higher CEC value. Meanwhile, the calcium ion exchange capacity of the prepared zeolite X sample is 248 mg/g, which is higher than that of commercial zeolites X (232 mg/g).

### 3.5. TG-DTA and XRF Analysis

**Figure 11:**
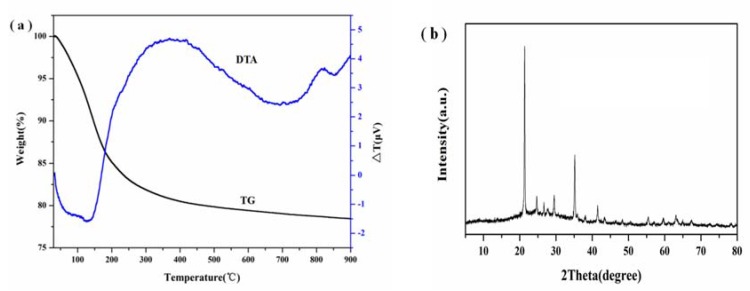
TG-DTA curves (**a**) of the prepared zeolite X sample at crystallization time and temperature, aging time, and temperature, H_2_O/Na_2_O and Na_2_O/SiO_2_ ratio of 5 h and 110 °C, 30 min and 30 °C, 40 and 1.4 and XRD pattern (**b**) of the prepared zeolite X sample after calcinating at 820 °C.

The chemical composition of the synthesized X zeolite is presented in Table 1. The synthesized X zeolite is composed of Si, Al, and Na. Meanwhile, the Si/Al ratio is 1.21, which agrees with the theoretical value of 1.13 in the raw material. In addition, the contents of Si in the raw material and the synthesized X zeolite is 3.22 and 2.88 g, respectively, which is a highly efficient use of diatomite. Thermal behavior of the obtained zeolite X sample was investigated using simultaneous TG/DTA thermoanalytical techniques. Typical TG/DTA thermograms for the prepared zeolite X sample in the temperature range of 25–900 °C are shown in Figure 11a. TG results showed that the synthesized zeolite X products lost all its moisture (22 wt %) at temperatures lower than 350 °C. In general, this kind of weight loss was due to the removal of water adsorbed on the zeolite surface and that present in the zeolite channels. Furthermore, DTA curves showed that the endothermic peaks occurred at lower temperatures (150 °C) of the synthesized zeolite X sample, which could be assigned to the loss of adsorbed water. However, the exothermic peaks at the temperature of 820 °C could be attributed to the framework collapse and crystallization of NaAlSiO_4_ (JCPDS 52-1342), which can be attributed to the nepheline (Figure 11b). The TG-DTA analysis indicated that the synthesized X zeolite possessed excellent thermal stability. 

### 3.6. N_2_ Adsorption Performance

**Figure 12:**
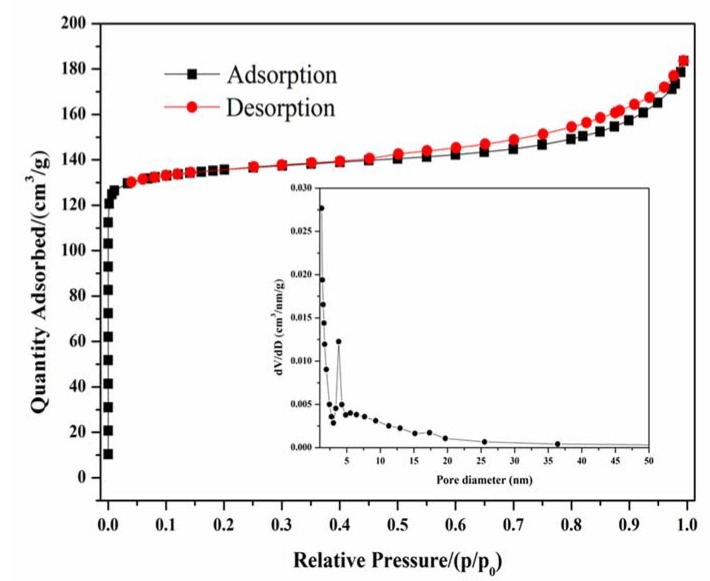
Nitrogen adsorption/desorption isotherms at 77 K of as synthesized zeolite X.

The N_2_ adsorption-desorption plot at 77 K for the prepared zeolite X is presented in Figure 12. Figure 12 shows a type I isotherm with the presence of steep nitrogen uptake at very low relative pressures (p/p_0_ = 0.03), which is attributed to the filling of micropores. Meanwhile, an obvious type H_4_ hysteresis loop (from 0.45 to 0.99) was observed, corresponding to the filling of uniform slit-shaped intercrystal mesopores, which was ascribed to the packing of zeolite crystals [35]. Thus, the zeolite X synthesized from diatomite possessed binary meso-microporous structure. The textural properties of the prepared zeolite X are summarized in Table 2. The specific surface area and total pore volume were up to 453 m^2^/g and 0.2838 cm^3^/g, respectively.

A comparison of the X zeolites prepared from different Si and Al sources are summarized in Table 3. The surface area of zeolite X prepared here is relatively high. Another comparison of different methods in the preparation of zeolites with diatomite as Si source is presented in Table 4. It can be seen from Table 4 that most methods need acid activation and calcination, which are not environmentally friendly and of high-cost. In addition, these conventional methods also take a longer time to achieve the transformation from minerals to zeolites. Therefore, the one-step hydrothermal method proposed in this research is more environmentally friendly and cheaper.

## 4. Conclusions

In this work, the zeolite X was obtained from diatomite via a simple hydrothermal method. In addition, the optimum preparation conditions of zeolite X were 5 h of crystallization time, 110 °C of crystallization temperature, 30 °C of aging temperature, 30 min of aging time, H_2_O/Na_2_O ratio of 40 and Na_2_O/SiO_2_ ratio of 1.4. The prepared pure zeolite X with binary meso-microporous structure possessed remarkable thermal stability, high calcium ion exchange capacity of 248 mg/g and large surface area of 453 m^2^/g. Furthermore, it is shown here that the calcium ion exchange capacity of the samples is mainly determined by their crystallization degree and higher crystallization degree means higher calcium ion exchange capacity. Compared with the traditional synthesis techniques, the hydrothermal process developed here is simple, low-cost, and environmentally friendly. In addition, the high purity of zeolite X using natural low-grade diatomite as raw material may be useful for potential industrial application as catalyst support or adsorbent.

## Figures and Tables

**Table 1:** The composition of the prepared zeolite X sample at crystallization time and temperature, aging time, and temperature, H_2_O/Na_2_O and Na_2_O/SiO_2_ ratio of 5 h and 110 °C, 30 min and 30 °C, 40 and 1.4.

Chemical Composition	SiO_2_	Al_2_O_3_	Na_2_O	Fe_2_O_3_	H_2_O	n_(Si/Al)_
Optimal X zeolite	35.31	24.87	15.17	0.96	22.90	1.21

**Table 2:** Textural properties of the zeolite X.

S_BET_ (m^2^/g)	S_micro_ (m^2^/g)	V_total_ (cm^3^/g)	V_micro_ (cm^3^/g)
453	399	0.2838	0.1866

**Table 3:** Comparison of different Si and Al sources on the surface areas of synthetic zeolite X.

Zeolite	Si and Al Source	BET Surface Area (m^2^/g)	Refs.
X	Fly ash	344	[36]
X	Feldspar	472	[19]
X	Bentonite	505	[19]
X	Kaolinite	591	[19]
X	Fly ash	404	[27]
X	Fly ash and sodium aluminate	397	[37]
X	Diatomite and aluminum hydroxide	453	This work

**Table 4:** Comparison of different methods in the preparation of zeolites with diatomite as Si source.

Zeolite	Al Source	Hydrothermal Time (h)	Synthenic Method	Refs.
Sodalite	/	/	Microwave heating methods	[38]
Y	Al_2_(SO_4_)_3_	6–48	H_2_SO_4_ activation and hydrothermal methods	[1]
ZSM-5	NaAlO_2_	40	Templation and hydrothermal methods	[39]
P	Aluminum hydroxide	6–24	Water-bathing methods	[40]
P	Paper sludge ash	24	Low-temperature methods	[41]
X	Aluminum hydroxide	5	Hydrothermal methods	This work
